# p27^kip1^ expression limits H-Ras-driven transformation and tumorigenesis by both canonical and non-canonical mechanisms

**DOI:** 10.18632/oncotarget.11656

**Published:** 2016-08-27

**Authors:** Ilenia Pellizzari, Linda Fabris, Stefania Berton, Ilenia Segatto, Francesca Citron, Sara D'Andrea, Martina Cusan, Sara Benevol, Tiziana Perin, Samuele Massarut, Vincenzo Canzonieri, Monica Schiappacassi, Barbara Belletti, Gustavo Baldassarre

**Affiliations:** ^1^ Division of Experimental Oncology 2, Department of Translational Research, CRO Aviano, National Cancer Institute, Aviano, Italy; ^2^ Pathology Unit, CRO Aviano, National Cancer Institute, Aviano, Italy; ^3^ Breast Surgery Unit, CRO Aviano, National Cancer Institute, Aviano, Italy; ^4^ Department of Experimental Therapeutics, M.D. Anderson Cancer Center, Houston, TX, USA

**Keywords:** p27kip1, stathmin, H-Ras and K-Ras, cell cycle progression, metastasis

## Abstract

The tumor suppressor protein p27^Kip1^ plays a pivotal role in the control of cell growth and metastasis formation.

Several studies pointed to different roles for p27^Kip1^ in the control of Ras induced transformation, although no explanation has been provided to elucidate these differences. We recently demonstrated that p27^kip1^ regulates H-Ras activity *via* its interaction with stathmin.

Here, using *in vitro* and *in vivo* models, we show that p27^kip1^ is an important regulator of Ras induced transformation. In H-Ras^V12^ transformed cells, p27^kip1^ suppressed cell proliferation and tumor growth *via* two distinct mechanisms: 1) inhibition of CDK activity and 2) impairment of MT-destabilizing activity of stathmin. Conversely, in K-Ras4B^V12^ transformed cells, p27^kip1^ acted mainly in a CDK-dependent but stathmin-independent manner.

Using human cancer-derived cell lines and primary breast and sarcoma samples, we confirmed in human models what we observed in mice.

Overall, we highlight a pathway, conserved from mouse to human, important in the regulation of H-Ras oncogenic activity that could have therapeutic and diagnostic implication in patients that may benefit from anti-H-Ras therapies.

## INTRODUCTION

The tumor suppressor protein p27^kip1^ (hereafter p27) was originally identified as a cyclin/CDK inhibitor, in particular of the CDK2-containing complexes[1, 2]. Subsequent studies demonstrated that it is also implicated in the regulation of several other biological activities, such as differentiation, apoptosis, motility and autophagy [1, 2].

Formal demonstration that p27 is a fundamental negative regulator of cell cycle progression with tumor suppressor properties primarily arose from the characterization of p27 knock-out mice[3-5]. Interestingly, most of the phenotypes of p27 null mice are reverted by the concomitant knock-out of the microtubules (MT)-destabilizing stathmin [6], demonstrating that p27/stathmin interaction plays a role in the control not only of cell motility [7-11] but also of cell proliferation[6, 12]. Mechanistically, we recently showed that p27 controls H-Ras-driven proliferation acting on its intracellular recycling and mono- bi-ubiquitination [12].

A large body of literature exists focusing on the cooperation between Ras and p27 during tumor onset and progression. It is widely accepted that p27 expression impacts on Ras-driven tumor progression. Interesting differences have been noted when p27 knock-out mice have been challenged with tumorigenic models relying on H-Ras *versus* K-Ras mutations. In tumors driven by K-Ras mutations, such as the urethane-induced [13, 14] or K-Ras^G12D^-induced [15] lung cancers, p27 acts as a haploinsufficient tumor suppressor gene and the loss of one allele is sufficient to induce the maximum oncogenic cooperation. Conversely, in H-Ras-driven tumorigenesis, such as the MMTV-H-Ras^V12^-induced breast and salivary gland cancers [16] or the DMBA/TPA skin carcinogenesis model [17], p27 acts as a classical tumor suppressor gene and loss of one p27 allele does not result in enhanced tumor growth. In these settings, many of the H-Ras^V12^/p27 null tumors displayed features characteristic of highly aggressive tumors [16]. Accordingly, it has been postulated that p27 controls cell H-Ras^V12^-driven transformation *via* not only the inhibition of the cyclin/CDK/RB pathway but also *via* RB-independent pathways [18].

We have recently observed that p27 participates to the regulation of H-Ras activity by modulating its recycling and ubiquitination [12]. Since H-Ras and K-Ras are differently regulated by recycling and ubiquitination [19, 20], it is expectable that p27 could differentially act in H-Ras *versus* K-Ras-driven tumor progression. Here, we address this hypothesis using different in *vitro* and in *vivo* model systems.

## RESULTS

### H-Ras efficiently transforms p27 null 3T3 fibroblasts

**Figure 1:**
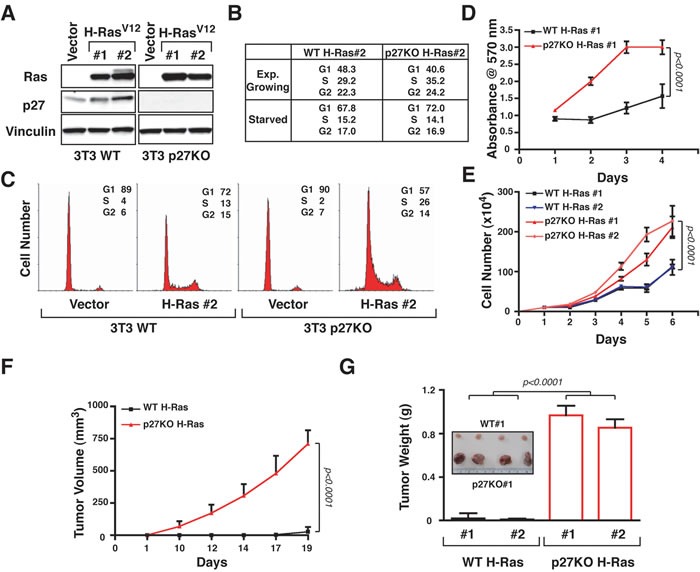
p27 null fibroblasts are much more sensitive to H-Ras^**V12**^-driven transformation than WT ones **A.** Western Blot analysis of H-Ras and p27 expression in two clones of 3T3 fibroblasts WT and p27KO, transfected with an empty vector (Vector) or H-Ras^V12^. Vinculin was used as loading control. **B.** Table reporting the data from FACS analysis of cell cycle distribution of WT and p27KO H-Ras^V12^ transformed fibroblasts in exponentially growing- or serum starved-conditions, as indicated. **C.** FACS analysis of cell cycle distribution of WT and p27KO control cells (vector) or H-Ras^V12^ transformed fibroblasts, collected after contact inhibition. **D.** Growth curves of WT and p27KO H-Ras^V12^ transformed fibroblasts over a four-days period, using the MTS assay. Values in the graphs represent the mean of three different experiments +/− SD. **E.** Growth curves of WT and p27KO H-Ras^V12^ transformed fibroblasts over a six-days period, using the Trypan blue exclusion test. Values in the graphs represent the mean of three different experiments +/− SD. **F.** Graph reports the *in vivo* growth of WT and p27KO H-Ras^V12^ transformed fibroblasts. Data represent the mean tumor volume (+/− SD) in 10 mice (5/each clone) injected with WT and 10 mice (5/each clone) injected with p27KO H-Ras^V12^ transformed fibroblasts and monitored for 19 days. **G.** Graph reports the mean tumor weight (+/− SD) from mice injected with WT (*n* = 5/cell clones) and p27KO H-Ras^V12^ transformed fibroblasts (*n* = 5/cell clones) and sacrificed after 21 days from the injection is reported. In the inset, a typical image of tumors removed from WT and p27KO H-Ras^V12^ transformed cells is shown. Significant differences (*p* value ≤ 0.05) are reported in the graphs and were calculated by Student's t-test.

We previously observed that p27 null cells and mice displayed higher Ras activity, due to different recycling and decreased mono-bi-ubiquitination [12]. To test if these differences may have any role in cell transformation, we generated and characterized H-Ras^V12^-transformed WT and p27KO 3T3 fibroblasts [7, 21]. Endogenous p27 expression was maintained in H-Ras^V12^ overexpressing-p27WT 3T3 cell clones (Figure 1A). WT and p27KO H-Ras cells were both less sensitive to serum deprivation than not transformed cells (Figure 1B and Supplementary Figure S1A). Yet, p27KO H-Ras^V12^ cells displayed increased S phase population when compared to WT H-Ras^V12^ cells, in exponential growth (Figure 1B) and after contact inhibition (Figure 1C). Furthermore, they entered into the cell cycle sooner than WT H-Ras^V12^ cells (Supplementary Figure S1A and B). Accordingly, in exponentially growing conditions p27KO H-Ras^V12^ cells had a significantly lower doubling time (Supplementary Figure S1C), and proliferated significantly more than the WT counterpart (Figure 1D and 1E).

In agreement with our previous observation [7, 8, 21, 22], WT *versus* p27KO-transformed cells displayed different ability to move, both in three-dimensional (3D)- (Supplementary Figure S1D) and in two-dimensional (2D)-extracellular matrix contexts (Supplementary Figure S1E), but not when 2D-migration was performed on plastic dish (Supplementary Figure S1F).

Subcutaneous injection of 1×10^6^ WT H-Ras^V12^ cells in nude mice (*n* = 5) were not sufficient to give rise to tumors, while the same number of p27KO H-Ras^V12^ cells determined the appearance of tumor masses within 10-12 days (data not shown). Using 2×10^6^ cells, all mice injected with WT H-Ras^V12^ cells developed slow growing tumors within 15-17 days, but p27KO H-Ras^V12^ cells formed fast growing tumors within 5-10 days (Figure 1F) as evidenced by differences in the explanted tumor masses (p < 0.0001, *n* = 5/clone), with no appreciable difference among the clones utilized (Figure 1G).

### p27/stathmin interaction regulates the metastatic phenotype of H-Ras^V12^-transformed fibroblasts

**Figure 2:**
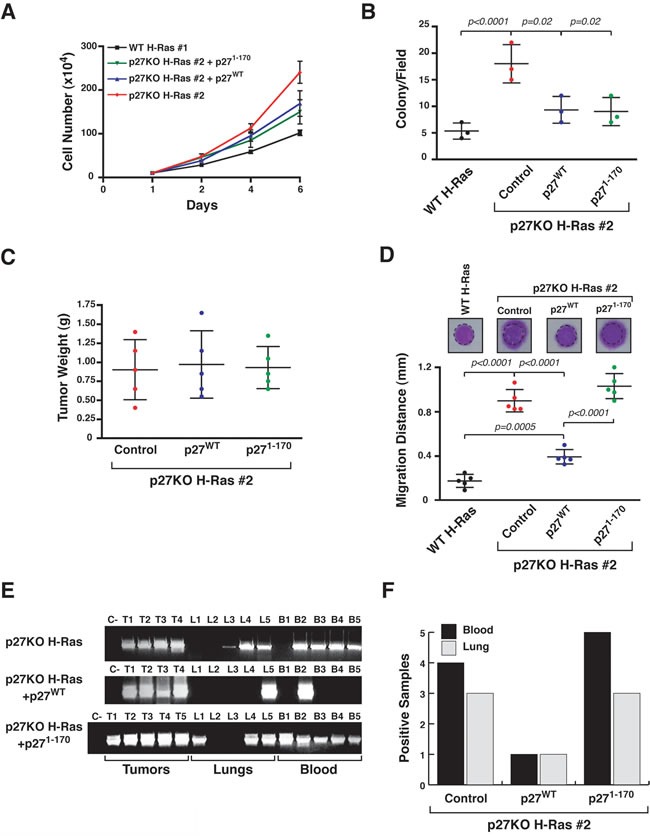
p27^WT^ reverts the metastatic ability of p27 null H-Ras^V12^ fibroblasts **A.** Growth curves of the indicated H-Ras^V12^ transformed fibroblasts over a six days period, using the Trypan blue exclusion test. Values in the graphs represent the mean of three different experiments +/− SD. **B.** Graph reports results from soft agar assay of the indicated H-Ras^V12^ transformed fibroblasts. At least 8 fields/well were counted. Data represent the mean (+/−SD) of three different experiments. **C.** Graph reports the mean tumor weight (+/− SD, *n* = 5/cell clones) from mice injected with the indicated H-Ras^V12^ transformed fibroblasts (1×10^6^), 30 days after the injection. **D.** Matrigel evasion assay of the indicated H-Ras^V12^ transformed fibroblasts. Cells were included in 3D-Matrigel, incubated in complete medium for 5 days and then stained with crystal violet. The mean distance (+/− SD) covered by the cells from the Matrigel-drops (5 drops/clone) is reported in the graph. A typical image of the stained drops is shown on the top of each column. The dashed line delimits the border of the drop. **E.** RT-PCR analysis of H-Ras^V12^ expression in RNA extracted from primary tumors (T), Lung (L) and Blood (B) from mice subcutaneously injected with the indicated cells. C- = Negative control of the PCR reaction. **F.** Graph reports the number of positive Blood and Lungs from mice described in (E) (5 mice/clone). Significant differences (*p* value ≤ 0.05) are reported in the graphs and were calculated by Student's t-test or Mann-Whitney t-Test as appropriate.

In line with our previous results showing that p27/stathmin interaction modulates H-Ras activity [12] we showed that p27^WT^ but not the p27^1-170^ mutant (unable to bind stathmin) [7-9, 12, 21] reduced ERK1/2 phosphorylation when reintroduced in p27KO cells (Supplementary Figure 2A). I*n vitro* experiments using growth curves (Figure 2A) and soft agar assays (Figure 2B) and *in vivo* experiments (Figure 2C) using subcutaneous injections, showed that p27KO H-Ras^V12^ fibroblasts re-expressing p27^WT^ or p27^1-170^ were barely (*in vitro*) or not (*in vivo*) affected in their growth by either p27^WT^ or p27^1-170^. However, p27^WT^, but not p27^1-170^, reduced the ability of p27KO H-Ras^V12^ transformed fibroblasts to move in 3D-Matrigel (Figure 2D) and to intravasate, extravasate and settle at distant sites in nude mice, as demonstrated by the presence of cells expressing the H-Ras^V12^ transgene in the blood and in the lungs of mice bearing subcutaneous tumors (5 mice/cell clone) (Figure 2E and 2F).

### Stathmin is necessary for the growth advantage of p27KO-H-Ras^V12^ fibroblasts

**Figure 3:**
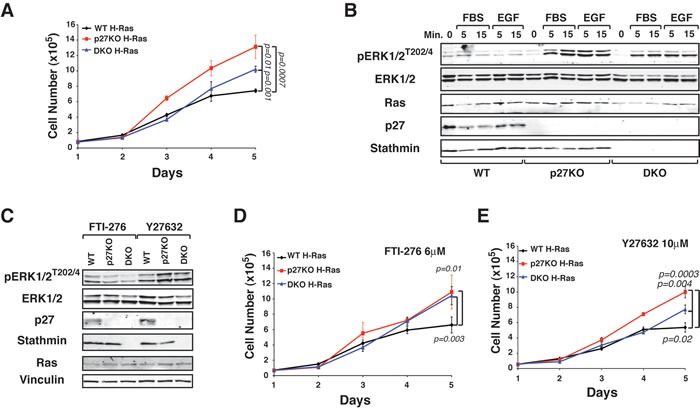
p27 controls H-Ras^V12^ activation via stathmin **A.** Growth curves of WT, p27KO and DKO H-Ras^V12^ transformed fibroblasts. Cells, plated on day 0, were counted at the indicated time points, by Trypan Blue exclusion test. **B.** Western Blot analysis of ERK1/2 activation in 3T3 WT, p27KO and DKO transformed with H-Ras^V12^, serum starved and then stimulated with 10% FBS (FBS) or EGF (3ng/ml), for 5 and 15 minutes, as indicated. The expression of total ERK1/2, H-Ras, p27 and stathmin is also reported. **C.** Western Blot analysis of ERK1/2 activation in the indicated H-Ras^V12^ transformed fibroblasts, following treatment with FTI-276 or Y277632 for 4 days. Expression of p27, stathmin and Ras is shown. Vinculin was used as loading control. **D.** and **E.** Growth curves of WT, p27KO and DKO H-Ras^V12^ transformed fibroblasts, treated with the Ras inhibitor FTI-276 (D) or the ROCK inhibitor Y27632 (E). Cells, plated on day 0, were counted at the indicated time points, by Trypan Blue exclusion test. Significant differences (*p* value ≤ 0.05) are reported in the graphs and were calculated by Student's t-test or Mann-Whitney t-Test as appropriate.

To test whether stathmin was involved, at least in part, in determining the different *in vitro* and *in vivo* growth observed in WT *versus* p27KO H-Ras^V12^ transformed fibroblasts, we used 3T3 fibroblasts derived from WT, p27KO and also double knock-out (DKO) for both, p27 and stathmin C57BL/6 embryos [12, 22]. Growth curve experiments confirmed that transformed p27KO cells grew much faster than WT cells and showed that DKO H-Ras^V12^ cells displayed an intermediate growth rate (Figure 3A) and western blot analyses demonstrated higher ERK phosphorylation in p27KO H-Ras^V12^ than in WT and DKO fibroblasts, both in basal conditions (Supplemental Figure S2A) and following serum (FBS) or Epidermal Growth Factor (EGF) stimulation (Figure 3B).

Upstream activators of ERK include the H-Ras-related small GTPase RhoA that is also regulated by p27 [22, 23]. RhoA signals to ROCK1 to control both cell proliferation and migration [24, 25]. To distinguish the effects of p27 on H-Ras and RhoA we pharmacologically inhibited H-Ras (FTI-276) or ROCK1 (Y27632). FTI-276 treatment abrogated the differences in ERK phosphorylation between the different genotypes, while Y27632 treatment did not (Figure 3C). When used in growth curve experiments, both inhibitors reduced the proliferation of H-Ras^V12^ transformed cells of all genotypes (Figure 3D and 3E). However, only FTI-276 inhibitor abrogated the differences in cell proliferation between p27KO and DKO cells (Figure 3D). Similar results were observed in soft agar assay experiments (Supplementary Figure S2B).

### p27 absence confers a growth advantage to H-Ras^V12^ but not K-Ras^V12^ transformed fibroblasts

**Figure 4:**
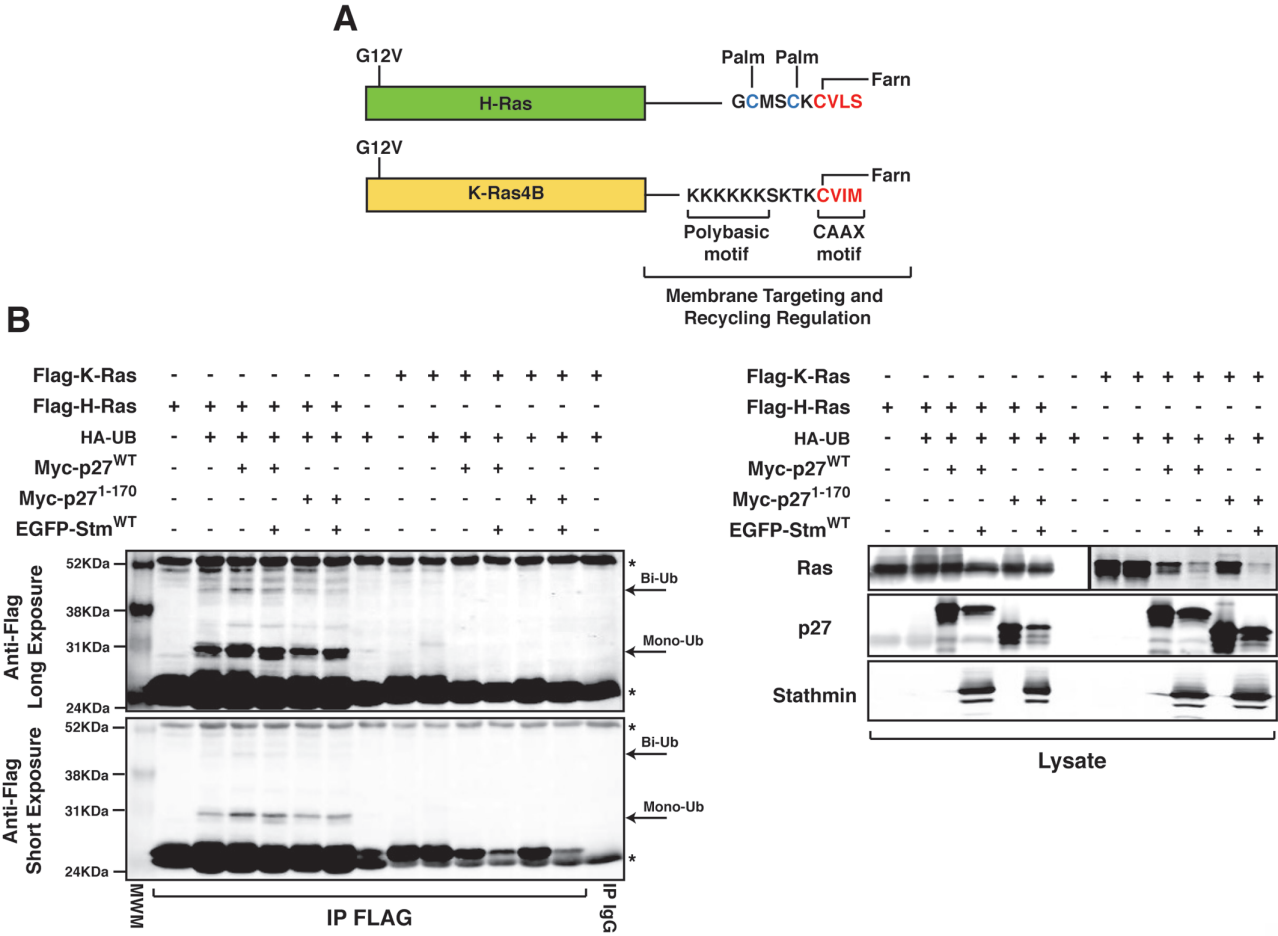
p27 does not control K-Ras4B^V12^ ubiquitination **A.** Schematic representation of H-Ras^V12^ and K-Ras4B^V12^ proteins, highlighting the differences in the C-terminal hypervariable region that governs their subcellular localization. Targeting to the membrane of H-Ras requires the farnesylation (Farn) of the CCAX motif and the palmitoylation (Palm) of C181 and C184. K-Ras membrane targeting is due to the CAAX farnesylation and the presence of the K175-K180 stretch (Polybasic region). The different C-terminus sequence and regulation results in the recycling of H-Ras but not K-Ras4B through the endosome compartment. Palm indicates the sites of palmitoylation; Farn indicates the sites of farnesylation. **B.** Immunoprecipitation analysis of FLAG-tagged H-Ras or K-Ras in 293T/17 cells, transfected with FLAG-tagged H-Ras (or K-Ras) in the presence or not of HA-tagged ubiquitin vector (HA-UB), Myc-tagged p27^WT^ or p27^1-170^ and EGFP-tagged stathmin expressing vectors, as indicated. Total cell lysates were immunoprecipitated with an anti-FLAG antibody and probed with anti-FLAG antibody, as reported in the figure. Arrows indicate mono- and bi-ubiquitinated forms of H-Ras. Arrowhead indicates unmodified Flag-tagged H-Ras (or K-Ras). Asterisks mark light and heavy IgG chains. Long and short exposures of the same blot are reported. In the lower panels, the expression levels of the transfected proteins in total protein lysates, using the indicated anti-Tags antibodies, are shown. N.B. The left part of this blot (i.e. the Flag H-Ras transfected cells) has been already published in *Fabris et al. Proc Natl Acad Sci USA* 112(45): 13916-21, *2015. doi: 10.1073/pnas.1508514112* and it is reported here only as a control for K-Ras ubiquitination.

These results, along with the data collected on normal fibroblasts [12], suggested that p27/stathmin interaction through the regulation of H-Ras/ERK activity partially controls H-Ras^V12^ driven transformation. If this was really the case, then no difference in cell transformation should be observed between p27KO and DKO fibroblasts transformed with K-Ras4B^V12^ oncogene. H-Ras and K-Ras4B (alternative splicing of exone 4 of K-Ras gene) are highly homolog proteins (83% aminoacid identity in the first 165 aminoacids) with an hyper variable C-terminus (24 aa) which comprises the membrane targeting sequence (Figure 4A) [26]. The different hyper variable region results in several unique features, such as their sub-cellular localization and their functional regulation. H-Ras and N-Ras, but not K-Ras4B, require recycling to be fully activated [19, 20] and H-Ras and N-Ras, but not K-Ras4B, are inhibited by mono-bi-ubiquitination [20]. In line with these notions, we observed that K-Ras4B^V12^ was not mono-bi-ubiquitinated either in the presence or absence of p27 and stathmin (Figure 4B).

**Figure 5:**
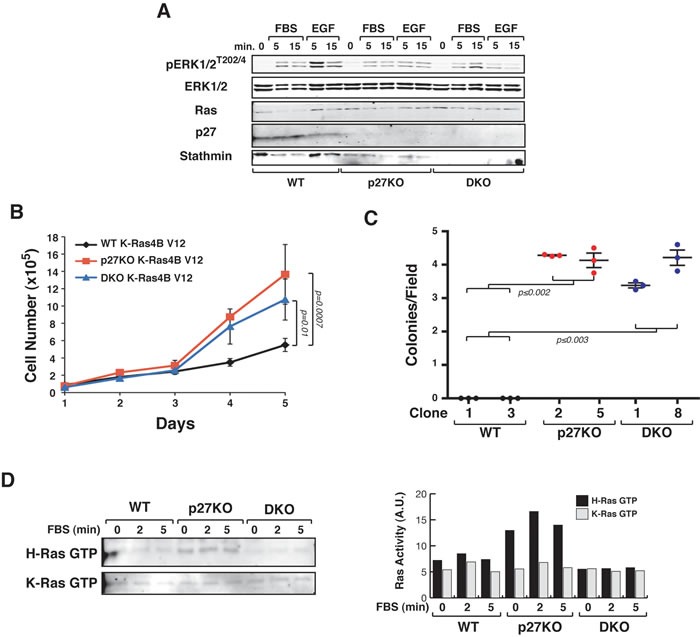
p27 does not control K-Ras4B^V12^ activation *via* stathmin **A.** Western Blot analysis of ERK1/2 activation in K-Ras4B^V12^ transformed WT, p27KO and DKO fibroblasts, serum starved and then stimulated with 10% FBS (FBS) or EGF (3ng/ml), for 5 and 15 minutes, as indicated. The expression of total ERK1/2, K-Ras, p27 and stathmin is also reported. **B.** Growth curves of the indicated K-Ras4B^V12^ transformed fibroblasts. Cells, plated on day 0, were counted at the indicated time points, by Trypan Blue exclusion test. Data represent the mean (+/− SD) of three different experiments performed in duplicate. **C.** Soft agar assay of the indicated K-Ras4B^V12^ transformed fibroblasts. Graphs show the quantification of colony number from three different experiments (+/− SD), considering at least 10 random fields for each clone. **D.** Pull down assay of GTP-bound H-Ras or K-Ras, as indicated, in MEFs of the indicated genotypes transformed with LgTAg and H-Ras^V12^ or K-Ras4B^V12^ and stimulated for 2-5 minutes with FBS. In the right graph, the activity of H-Ras or K-Ras is reported. In each graph, statistical significance is calculated by Student's t-test and expressed by a p value ≤ 0.05 (ns, not significant). A.U., arbitrary units.

K-Ras4B^V12^ p27KO and DKO transformed cells displayed similar levels of ERK1/2 phosphorylation (Figure 5A) and proliferated fairly at the same extent but significantly faster than WT cells (Figure 5B). No substantial difference in the transformation efficiency was detectable by soft agar assay between p27KO and DKO K-Ras4B^V12^ cells (Figure 5C). We next analyzed, by pull down assay, the levels of GTP-bound active Ras proteins in WT, p27KO and DKO cells transformed with H-Ras^V12^ or with K-Ras4B^V12^, in basal conditions and after stimulation with serum. As expected all transformed cells displayed constitutively active Ras, whose activity only slightly increased upon serum stimulation (Figure 5D). However, basal and serum-stimulated H-Ras^V12^ (but not K-Ras4B^V12^) activities were significantly higher in p27KO when compared to WT and DKO transformed cells (Figure 5D), supporting the possibility that regulation of H-Ras ubiquitination by p27 and stathmin participated in the control of its activity.

### Human and mouse p27 equally inhibit cell motility of H-Ras^V12^-transformed cells

In all experiments reported above, we consistently observed that both H-Ras^V12^ and K-Ras^V12^ transformed immortalized 3T3 WT cells with very low ability, especially when used below passage 45 (Figures 1, 2, 3, 4, 5). This observation pointed that the p27/CDK/RB pathway plays a fundamental role as gatekeeper from Ras-induced cell transformation. Our results also showed that this function cannot be fully rescued by the reintroduction of human p27^WT^ in p27KO transformed cells. To exclude species-specific effects due to the use of human p27 (h-p27^WT^) in rescue-experiments, we re-performed some of the same assays in p27 null H-Ras^V12^ transformed cells, reintroducing the mouse p27, either wild type (m-p27^WT^) or mutated in the binding to Cyclin/CDKs complexes [8, 12, 27] (m-p27^CK-^) (Supplementary Figure S3A and B). In line with the results obtained with the human protein, m-p27^WT^ and mp27^CK-^ proteins only slightly affected the ability of H-Ras^V12^ transformed cells to grow in culture or in soft agar (Supplementary Figure S3C and D) but significantly reduced cell motility (Supplementary Figure S3E).

### p27/stathmin interaction regulates H- Ras^V12^- but not K-Ras^V12^-driven transformation, *in vivo*

**Figure 6:**
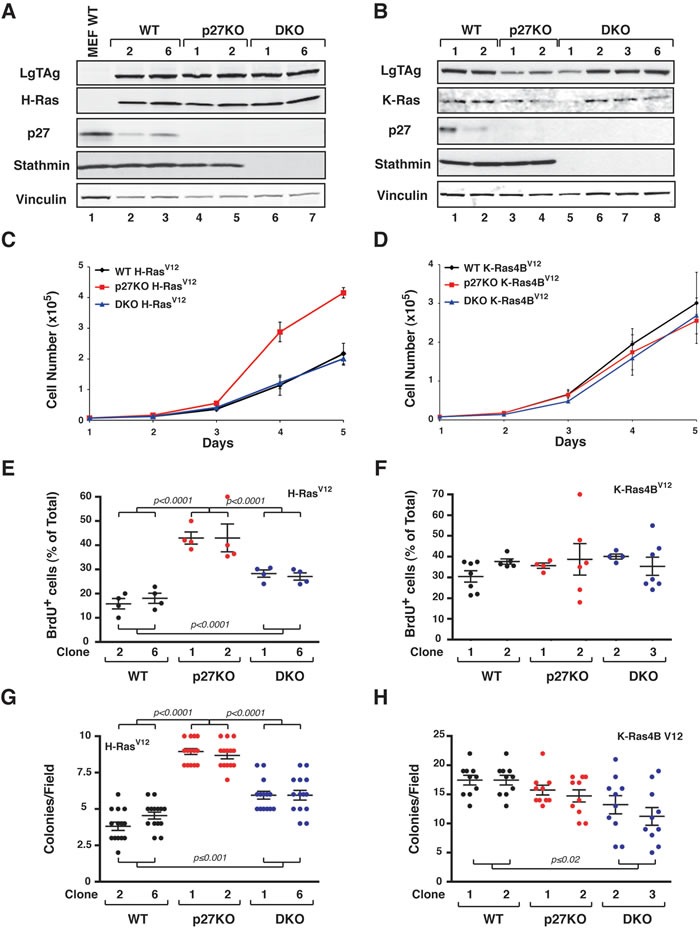
H-Ras^V12^ but not K-Ras4B^V12^ confers a growth advantage to p27 null MEFs, *in vitro* **A.** and **B.** Western Blot analysis of primary MEF WT, p27KO and DKO transduced with LgTAg and H-Ras^V12^ (A) or K-Ras4B^V12^ (B). The expression levels of the exogenous proteins LTAg, H-Ras and K-Ras are reported, as well as the endogenous expression of p27 and stathmin. Vinculin was used as loading control. **C.** and **D.** Growth curves of H-RasV12 (C) and K-Ras4BV12 (D) -transformed MEF. Two independent clones for each genotype were tested. Cells, plated on day 0, were counted at the indicated time points, by Trypan Blue exclusion test. Data represent the mean (+/− SD) of three different experiments performed in duplicate. **E.** and **F.** Graphs report the quantification of BrdU positive cells in exponentially growing H-Ras^V12^ (E) and K-Ras4B^V12^ (F) -transformed MEF. Data are expressed as percentage of total cell number. **G.** and **H.** Soft agar assay of H-Ras^V12^ (G) and K-Ras4B^V12^ (H) -transformed MEFs. Graphs show the quantification of colony number from three different experiments (+/− SD), considering at least 10 random fields for each clone. In each graph, statistical significance is calculated by Student's t-test and expressed by a p value ≤ 0.05. Significant differences are reported in the graphs.

To avoid the possible bias due to clonal selection and in the generation of 3T3 cells we next concomitantly transduced with and SV40 Large TAg (LgTAg) and with H-Ras^V12^ (Figure 6A) or K-Ras4B^V12^ (Figure 6B) primary MEF of the different genotypes. Since SV40 LgTAg oncogene simultaneously inactivates p53 and RB, we anticipated that in this model the relevance of CDK-inhibition by p27 would be less pronounced.

In this model p27KO transformed with LgTAg and H-Ras^V12^ grew at higher extent than the correspondent WT and DKO cells both in culture and in anchorage independent manner (Figure 6C, 6E and 6G). Correspondent cell clones transformed with K-Ras4B^V12^ proliferated all at similar level, in the presence or absence of p27 (Figure 6D, 6F and 6H).

**Figure 7:**
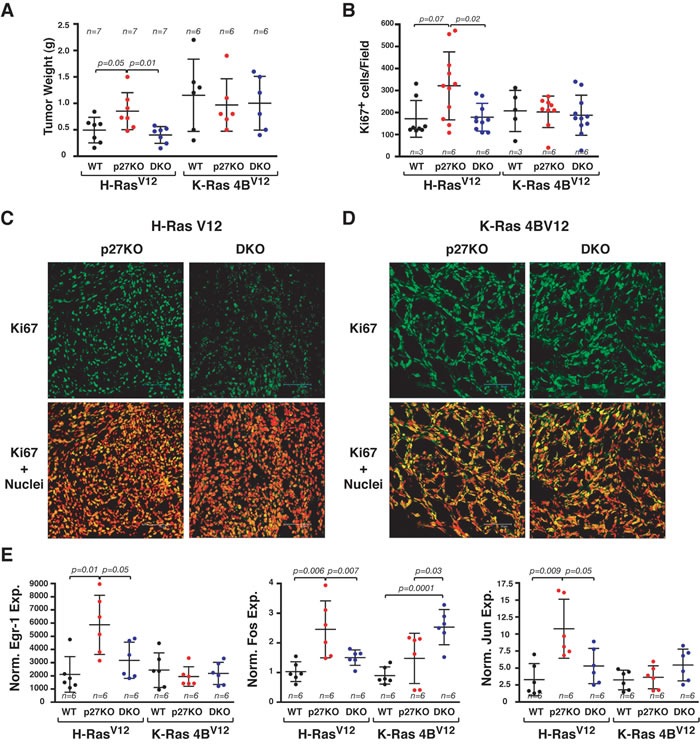
H-Ras^V12^ but not K-Ras4B^V12^ confers a growth advantage to p27 null MEF, *in vivo* **A.** Graph reports the tumor weight of H-Ras^V12^ or K-Ras4B^V12^ transformed MEF LTAg, subcutaneously injected in nude mice. The number of tumors/each cell clone is indicated on top of the graph. **B.** Graph reports the quantification of Ki67 positive cells, expressed as positive cells for field, in the tumors described in (A). Number of tumors analyzed is reported in the graph. **C.** and **D.** Representative images of Ki67 staining of tumors described in tumors derived from H-Ras^V12^ (C) and K-Ras4B^V12^ (D) transformed MEF. In the upper panels, confocal images of the cells stained for Ki67 (AlexaFluor488, pseudocolored in green) are shown. In the lower panels, the merging of Ki67 and nuclei (propidium iodide, pseudocolored in red) is displayed. Bar is 75 μm. **E.** qRT-PCR of EGR-1, Fos and Jun expression in RNA extracted from the same tumors described in (A). Gene expression was normalized using two housekeeping genes. The number of tumors/each cell clone analyzed is indicated in the graph. In each graph, statistical significance is calculated by Student's t-test and expressed by a p value ≤ 0.05. Only significant differences are reported in the graphs. Data are represented as mean ± SD. A.U., arbitrary units.

Similarly, tumors from LgTAg/H-Ras^V12^ transformed p27KO MEF had significantly higher volume (Figure 7A) and number of Ki67 positive cells (Figure 7B and 7C), when compared to both WT and DKO counterparts. Conversely, LgTAg/K-Ras4B^V12^-transformed MEFs of all three genotypes displayed similar tumor volume (Figure 7A) and Ki67 expression (Figure 7B and 7D). In explanted tumors, the expression of the Ras-ERK downstream targets Egr-1, c-Fos and Jun-B was higher in p27KO cells only in the presence of H-Ras^V12^ (Figure 7E).

### p27/stathmin interaction controls MAPK activation in human tumors

**Figure 8:**
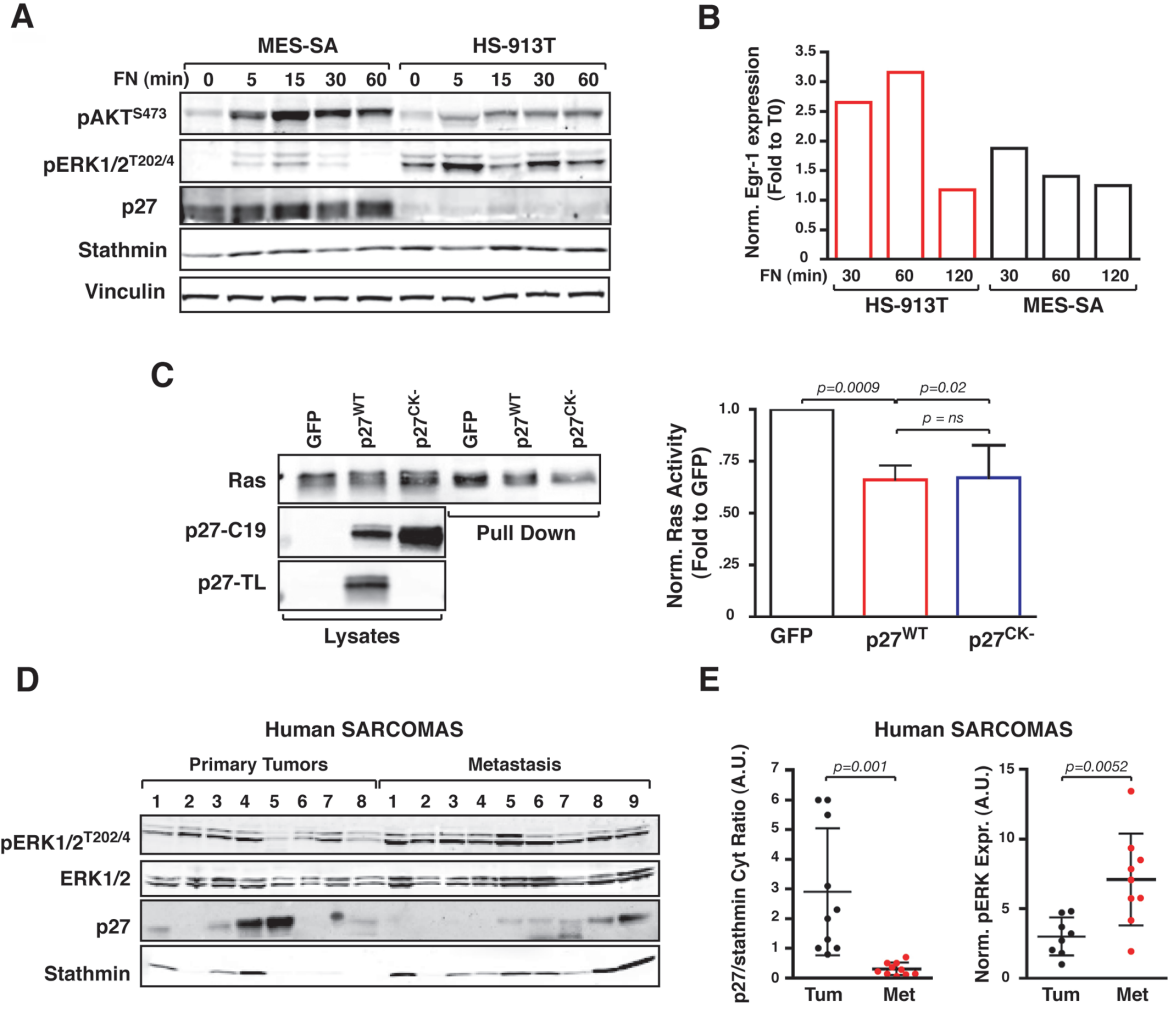
Low cytoplasmic p27/stathmin ratio is associated with hyper-activation of ERK1/2 pathway in human sarcoma and sarcoma-derived cell lines **A.** Western Blot analysis of ERK1/2 and AKT phosphorylation status in human fibrosarcoma cell lines adhered to Fibronectin (FN) for the indicated time, following serum starvation. Expression of p27 and stathmin is shown. Vinculin was used as loading control. **B.** qRT-PCR analysis of Egr-1 expression in human fibrosarcoma cell lines adhered to Fibronectin (FN) for the indicated time, following serum starvation. Gene expression was normalized using two housekeeping genes. Data are expressed as fold-increase over the time zero point. **C.** Pull down assay of Ras activity in HT-10180 cells overexpressing or not the p27^WT^ or the p27^CK-^ proteins, as indicated. The blot was incubated with two different anti-p27 antibodies to appreciate the expression of the WT and CK- proteins. p27-C19 directed against the protein C-terminus that recognizes both the WT and the CK- protein. p27-TL directed against the CDK-binding domain recognizes only the WT protein. Graph on the right reports the normalized Ras activity, expressed as fold increase over the control-transfected cells, representing the mean (+/− SD) of three different experiments. **D.** Western Blot analysis of ERK1/2 phosphorylation, p27 and stathmin expression in the cytoplasmic fractions of human primary (*n* = 8) and metastatic (*n* = 9) sarcoma samples. **E.** Graph reports quantification of Western Blot analysis in (D) of the ratio of cytoplasmic p27/stathmin levels (left graph) and of phospho-ERK1/2 expression (right graph). In each graph, statistical significance is calculated by Student's t-test and expressed by a *p* value ≤ 0.05. Data are represented as mean +/− SD. A.U., arbitrary units.

To evaluate the relevance of our findings in human cancer, we chose the soft tissue sarcomas (STS) as human counterpart of transformed fibroblasts. Following proliferative stimuli, MES-SA cells (established from primary STS) displayed high levels of p27 coupled with low levels of ERK1/2 activation and EGR1 expression, while HS-913T cells (established from metastatic STS) displayed low/null p27 levels, high ERK1/2 activation and EGR1 expression (Figure 8A and 8B). Using HT1080 cells, (a STS cell line harboring the N-Ras^Q61K^ mutation and a well-known model for Ras-driven transformation [28]) we next shown that Ras activity was reduced by one third by the overexpression of p27^WT^ or p27^CK-^ (Figure 8C). Since N-Ras shares the same recycling- and ubiquitin-mediated regulation of H-Ras [19, 20], our result supported the hypothesis p27 could control H/N-Ras activity in human STS. Accordingly, in a panel of already characterized human sarcoma specimens [7], low cytoplasmic p27/stathmin ratio was significantly associated with high levels of ERK1/2 phosphorylation (Figure 8D) and, when sarcoma specimens were segregated in primary *vs* metastatic tumors, we observed that metastatic tumors specimens displayed a significant lower p27/stathmin ratio coupled with a significantly higher ERK activation (Figure 8E).

Finally, using BRAF-mutated colorectal carcinoma cells (i.e. Colo-201 and Colo-205 in Supplementary Figure S4A and B) and HER2-overexpressing mammary carcinoma cells (i.e. SK-BR-3 and MDA-MB-453 in Supplementary Figure S4C), we consistently detected an inverse correlation between p27/stathmin cytoplasmic ratio and the activation of ERK1/2 and the expression of EGR-1 (Supplementary Figure S4A-C). Importantly, knock-down of p27 in SK-BR-3 cells increased ERK1/2 phosphorylation (Figure S4D) and low cytoplasmic p27/stathmin ratio was significantly associated with a higher level of ERK1/2 phosphorylation in primary breast carcinomas (Supplementary Table S1 and Supplementary Figure S4E).

## DISCUSSION

The results presented in this manuscript highlight several interesting features of the tumor suppressor gene p27 in the control of cell Ras-induced cell transformation and metastasis formation.

The most salient observation regards the susceptibility of WT or p27-null immortalized fibroblasts to the transforming activity of H-Ras^V12^ and K-Ras^V12^. The fact that p27-null fibroblasts are more prone to transformation and that reintroduction of p27 expression is not able to fully revert their phenotype reinforces the concept that control of CDK activity by p27 represents a significant barrier against cell transformation. The combined use of Large T-antigen and H-/K-Ras^V12^ (Figures 5 and 6) further confirms the relevance of p27 in controlling CDK activity during cell transformation. Yet, once cells are transformed by Ras, re-expression of p27 limited cell motility but failed to properly control *in vitro* proliferation and *in vivo* growth. Since we were concerned by the possibility that accumulation of concomitant mutations and/or genetic alterations could somehow affect the phenotype of p27KO H-Ras^V12^ cells, we repeated our experiments using 2 additional models (i.e. C57Bl6 3T3 and primary MEFs transformation), overall confirming the observation made using the Sv129 3T3 cells (Figures 1 and 2). It is interesting to note that in the case of v-Src transformed cells, reintroduction of the degradation resistant p27^T187A^ mutant, completely reverted the phenotypes of p27-null cells [21], suggesting that the pathways activated by Ras^V12^ and v-Src differently impact on the tumor suppressor roles of p27. Yet, one limitation of this study resides in the difficulty to discern whether one, or more of the multiple functions of p27, in the control of CDKs activities, MT-stability, actin reorganization, gene transcription and mitotic division [1, 2] could render p27 null cells more prone to transformation independently from the oncogene used.

A second interesting finding is the different potential displayed by p27 in restraining H-Ras^V12^ *versus* K-Ras^V12^-4B induced transformation. Only in H-Ras^V12^ transformed cells p27 controls not only CDKs activation but also Ras activity/localization *via* stathmin. Consequently, p27-null cells had a further advantage in cell growth and invasion that is likely dependent by the lack of feedback control of Ras exerted by p27 when located into the cytoplasm. It is interesting to note that a role for cytoplasmic p27 in the inhibition of Ras activity, *via* GRB2 binding, was previously reported [29]. Yet, it was not specified whether H- or K-Ras activity was tested. p27 expression was also reported to be necessary to mediate the inhibition of H-Ras-induced transformation induced either by STAT1 or by dominant negative Rho, likely in a RB-independent or partially dependent manner [18, 30]. More recent evidences demonstrated that in MEFs and in urinary bladder HT1197 cells, carrying a N-Ras Q61R mutation, p27 inhibits cell motility likely by reducing ERK activation [31], independently supporting our findings. Overall these data point to p27 as a relevant regulator of Ras activity in a cytoplasmic and CDK-independent manner.

Our work presents the limitation due to the use of constituvely active Ras mutant vectors. It has to be considered that the K-Ras4B gene used in this work is an alternatively spliced version of the K-Ras gene that could represent only a minor portion of the total K-Ras transcribed, as we observed in a model of skin carcinogenesis [40]. Thus, our model could not fully recapitulate the activation of endogenous K-Ras that is transcribed as K-Ras4A and K-Ras4B, with the former still subjected to recycling to be fully activated [19, 20].

The role(s) played by p27 when located in the cytoplasm are at the center of an interesting scientific debate. It has been considered either a cellular *escamotage* to inactivate nuclear p27 [1, 32] or a more complex way to control other signaling pathways and processes [12, 23, 29], such as cell death and autophagy [33, 34] or cell motility [7, 23]. Most of these activities have been attributed to the C-terminal portion of p27, containing several important regulatory elements. It is interesting to note that, at least in MEN syndrome [35], breast cancer [36, 37], intestine neuroendocrine tumors [38] and prostate cancer [39], the CDKN1B gene (encoding for p27) is frequently mutated in the C-terminal portion of the protein [36-39]. One of the mutations, repeatedly identified in human breast cancers, is the E171^®^Stop [36, 37] that results in a truncated protein (p27^1-170^) that we have characterized here and in previous publications [7-9, 12, 21].

Our data show that p27 and stathmin regulate in concert H-Ras but not K-Ras activity. This evidence could explain the contradictory tumor suppressor roles ascribed to p27 in different mouse models of cancer. In the presence of K-Ras mutations, p27 acts invariably as a haploinsufficient tumor suppressor gene [13-15]. In the presence of H-Ras, the complete loss of p27 is necessary to favor tumor growth in mice [16, 17], suggesting that the presence of one p27 allele is still able to restrain cell proliferation. According to this model, the fact that Ras-MAPK pathway activation results in phosphorylation of S10 and cytoplasmic delocalization of p27, likely represents a feedback control loop of particular importance to prevent unwanted cell cycle entry when the mitogenic extracellular stimuli are below the required threshold.

High levels of stathmin expression have often been linked to the acquisition of a metastatic phenotype [41]. The use of knock-out mice and cells allowed us to exclude a primary role of stathmin in the onset of several type of primary tumors in mice [40], suggesting that the function here described for p27/stathmin interaction can be unmasked in normal mice, cells and tissues only in the context of p27 absence. This concept may be of particular relevance in human tumors where p27 levels and localization are finely regulated and may also explain the contradictory results reported so far for stathmin in the control of cell growth, when gain-of-function [42, 43] or knock-out [44, 45] models were considered.

Successful targeting of the Ras-MAPK pathway represents a promising tool to treat aggressive human cancers, but optimal selection is needed to identify patients who would benefit from such therapies. Our findings suggest that evaluation of p27 and stathmin expression may contribute to this selection. This is particularly relevant for breast cancer, where Ras oncogenes are infrequently mutated but often hyperactive [46] and p27 mutations could be driver oncogenic events [36, 37].

## MATERIALS AND METHODS

Detailed description of the material and methods used is provided in supplementary material.

### Study approval

Sarcoma and breast cancer tissues were collected at CRO Aviano, Italy and stored in the Institutional Biobank, provided that the specific informed consent was obtained from the patient. Scientific use of biological materials was approved by the Internal Review Board (IRB) of CRO Aviano (# IRB-07/2015).

All animal experimentation were reviewed and approved by the CRO institutional Animal Care and Use Committee (OPBA), authorized by Italian Ministry of Health (# 616/2015-PR) and conducted according to that OPBA's guidelines.

### *In vivo* experiments

Primary tumors were established by subcutaneous injection of 1×10^6^ or 2×10^6^ transformed cells into the flanks of female athymic nude mice (Harlan, 7-8 weeks of age). Tumor growth was monitored every other day for up to 26 days from injection.

### Cell cultures and generation of stable cell clones

Primary wild type (WT), p27 knock-out (p27KO) and p27/stathmin double KO (DKO) mouse embryo fibroblasts (MEF) were prepared from embryos at day 13.5, according to standard procedures [6, 7]. 3T3 fibroblasts were generated from primary MEFs, as described [47]. MEF, 3T3 fibroblasts, 293T/17, HEK 293, MDA-MB-453 and SK-BR-3 human mammary adenocarcinoma cells and MES-SA, HS-913T, HT-1080 sarcoma cell lines, were all cultured in DMEM supplemented with 10% FBS (Sigma). Colo-201 and Colo-205 human colorectal adenocarcinoma cells were cultured in RPMI-1640 supplemented with 10% FBS (Sigma).

3T3 fibroblasts were transformed using H-Ras^V12^ (gently provided by Dr. R. Baserga) and K-Ras4B^V12^ (from ADDGENE, donated by Dr. T. Jacks) cDNAs both cloned in pMSCV-hygro retroviral vector (Clontech). Primary MEFs were transformed using concomitantly SV40 Large TAg (provided by Dr. R. Maestro) and pMSCV-Hygro-H-Ras^V12^ or pMSCV-Hygro-K-Ras4B^V12^. Human p27^WT^ or p27^1-170^ were described [7, 21, 22]; mouse p27^WT^ and p27^CK-^ cDNAs were provided by Dr. Bruno Amati.

### Proliferation and motility assays

Proliferation assays include: growth curve experiments, using the Trypan Blue exclusion test and the MTS assay (Promega); cell cycle distribution using flow cytometry; BrdU incorporation assay (Roche); soft agar assays; and tissues staining with Ki67.

Motility assays include were performed essentially as described [7, 21, 22] and include 3D-Matrigel™ evasion assay ; transwell-based migration assay using HTS Fluoroblok™ coated with 20μg/ml fibronectin (Sigma) and wound-healing assay.

### Tissue samples

A total of 17 sarcoma (leiomyosarcomas and fibromyosarcomas) and 37 breast tumor specimens were collected and diagnosed at Centro di Riferimento Oncologico (CRO) of Aviano (Italy), according to the World Health Organization (WHO) criteria. Sarcoma samples were described elsewhere [7] and derived from primary (*n* = 8) or metastatic samples (*n* = 9). Breast cancer specimens derived from locally advanced primary tumors, as better specified in Supplementary Table 1.

## SUPPLEMENTARY MATERIAL FIGURES AND TABLE


