# Supplementary material for: p27kip1 expression limits H-Ras-driven transformation and tumorigenesis by both canonical and non-canonical mechanisms
Source: Oncotarget. 2016 Aug 27;7(40):64560–74. doi: 10.18632/oncotarget.11656 (PMC5323099; doi:10.18632/oncotarget.11656)
Supplement: Supplementary file 1 [file oncotarget-07-64560-s001.pdf]

# p27$^{kip1}$ expression limits H-Ras-driven transformation and tumorigenesis by both canonical and non-canonical mechanisms

## Supplementary Material

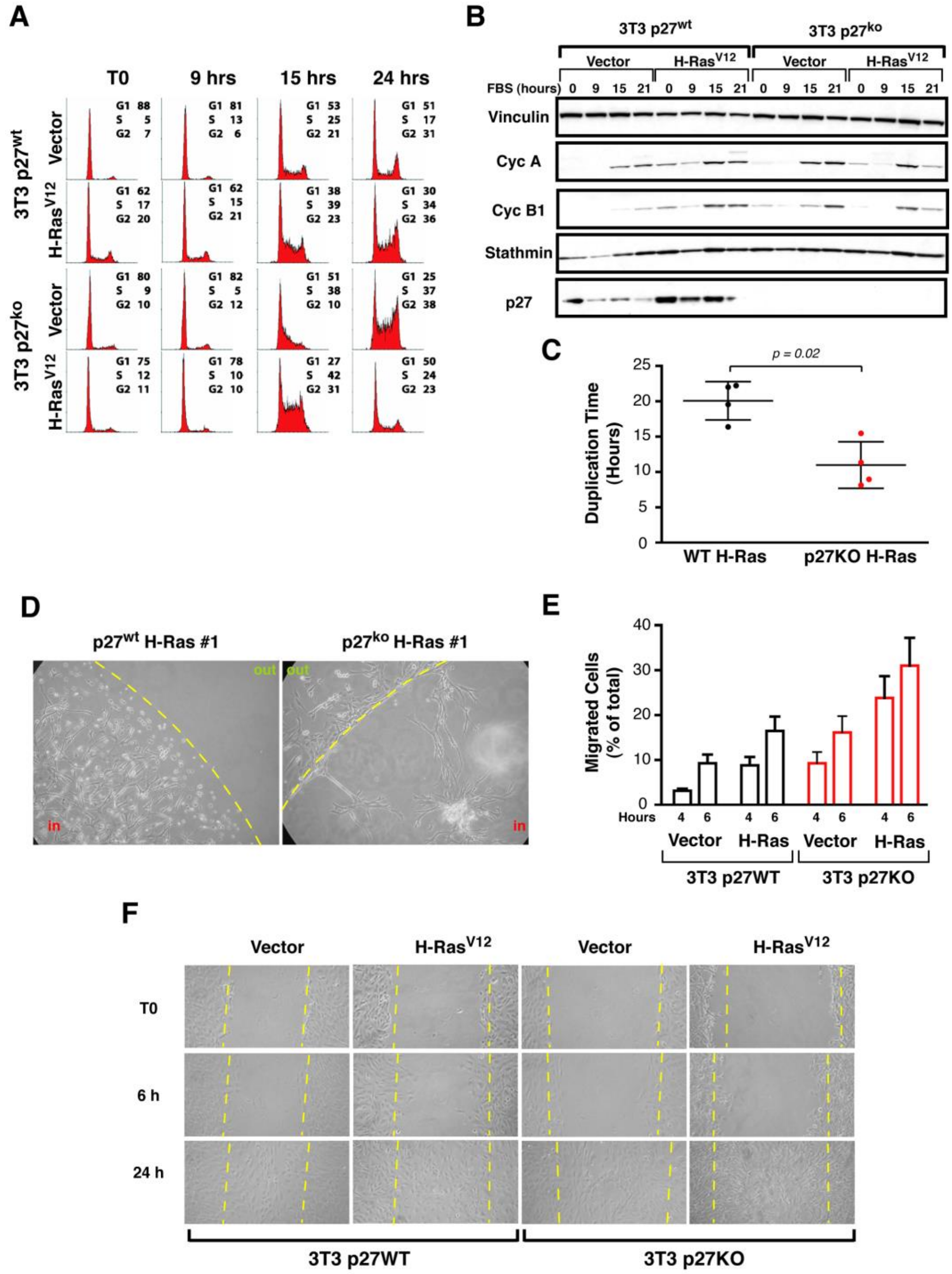


**Supplementary Figure S1.** *p27KO H-Ras$^{V12}$ fibroblasts display faster cell cycle entry and increased motility.*

**A** FACS analysis of typical cell cycle distribution observed in WT and p27KO control cells (vector) or H-Ras$^{V12}$-transformed fibroblasts, serum starved (T0) and then released in complete medium for the indicated times.

**B** Western Blot analysis of Cyclin A (Cyc A), Cyclin B1 (Cyc B1), stathmin and p27 in WT and p27KO control (Vector) or H-Ras$^{V12}$-transformed fibroblasts, serum starved (0) and then released in complete medium (FBS) for the indicated times. Vinculin was used as loading control.

**C** Graph reports the duplication time of WT and p27KO H-Ras$^{V12}$-transformed fibroblasts as evaluated in exponentially growing condition over a period of 4 days. Data report the mean of 2 ± SD of two different experiment performed with two different clones/genotype. Significance was calucluated using the Mann-Whitney unpaired test.

**D** Matrigel evasion assay. Cells were included in a 3D-matrigel drop and incubated in complete medium for 48 hours. A typical image in phase contrast microscopy, using a 10X objective, is shown. Cells still included in the Matrigel drop (In) and cells already exited (Out) are indicated.

**E** Transwell migration assay of WT and p27KO control cells (vector) or H-Ras$^{V12}$-transformed fibroblasts plated on transwell inserts coated with fibronectin and allowed to migrate up to six hours. Data represent the mean of 2 experiments performed in duplicate.

**F** Wound healing migration assay of WT and p27KO control cells (vector) or corresponding H-Ras$^{V12}$-transformed fibroblasts, grown to confluence on plastic dishes and then scratched and allowed to migrate into the wound up to 24 hours. Typical images in phase contrast microscopy, using a 10X objective, are shown.

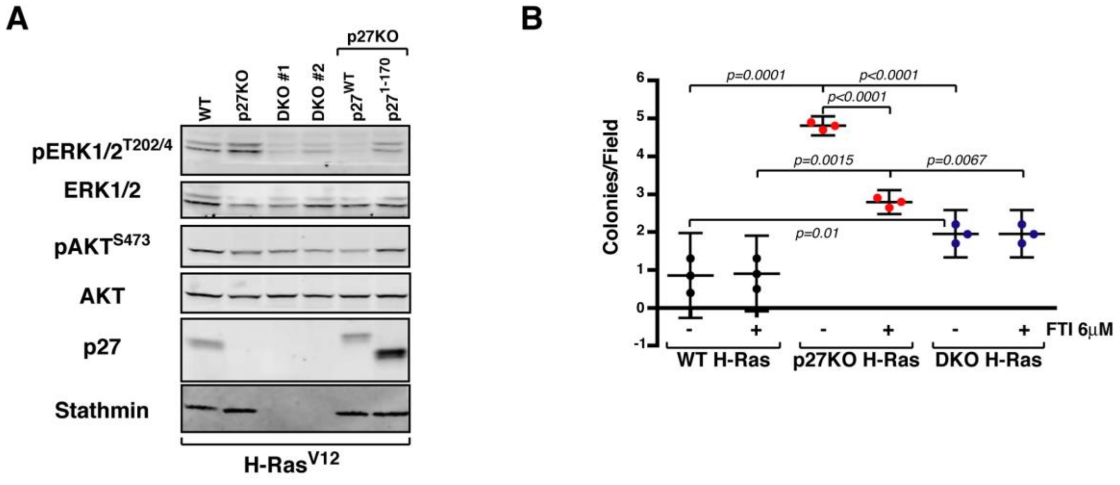


**Supplementary Figure S2.** *p27/stathmin interaction regulates H-Ras activity*.

**A** Western Blot analysis of ERK1/2 activation in 3T3 WT, p27KO and DKO transformed with H-Ras$^{V12}$ and in p27KO transformed fibroblasts re-expressing p27$^{WT}$ or p27$^{1\text{-}170}$ (unable to bind stathmin) proteins. The expression of total ERK1/2, phosphorylated and total AKT, p27 and stathmin is also reported.

**B** Soft agar assay of the indicated H-Ras$^{V12}$ transformed fibroblasts, treated or not with FTI-276 inhibitor. Data represent the mean (+/- SD) of three different experiments in which colonies were counted in 8-10 fields/well for each condition.

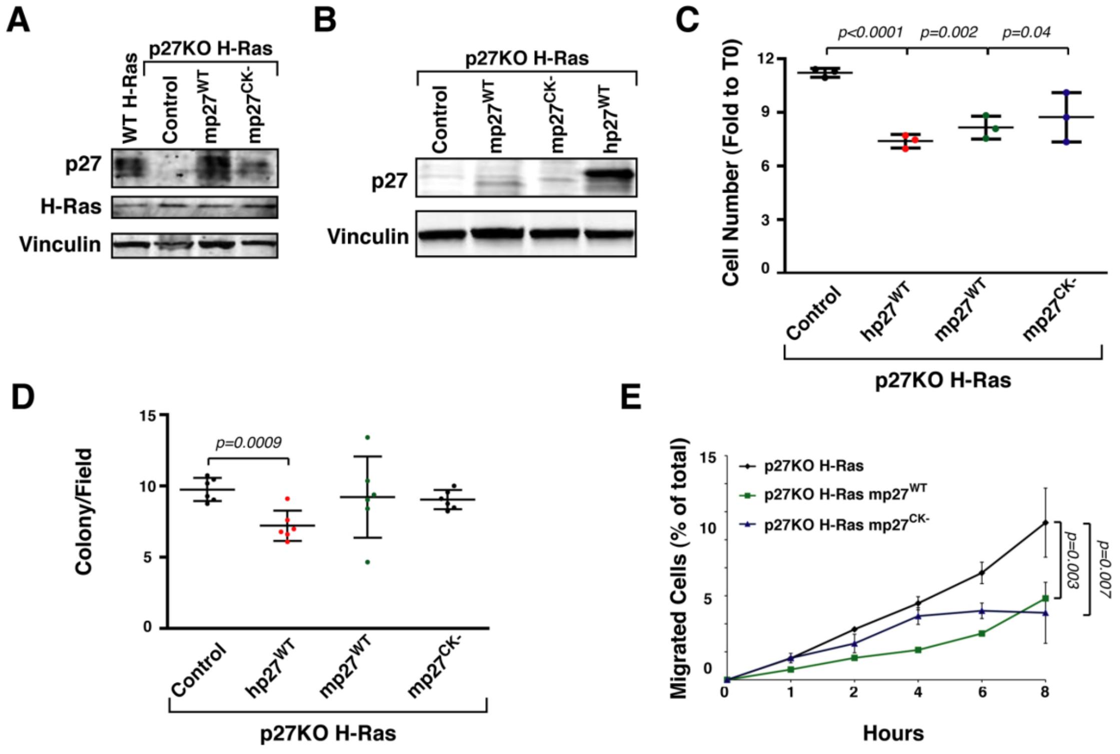


**Supplementary Figure S3.** *Re-expression of human or mouse p27 proteins in p27 null H-Ras$^{V12}$ fibroblasts have an equal impact on their phenotypes.*

**A** Western Blot analysis of p27 and Ras expression in the indicated p27KO H-Ras$^{V12}$ cell clones expressing mouse p27$^{WT}$ or p27$^{CK-}$ (mp27$^{WT}$ or mp27$^{CK}$) proteins. Vinculin was used as loading control. WT H-Ras$^{V12}$ cells were used as control of endogenous p27 expression.

**B** Western Blot analysis of p27 expression in the indicated p27KO H-Ras$^{V12}$ clones expressing mouse (mp27$^{WT}$ or mp27$^{CK}$) or human p27 (hp27$^{WT}$) proteins. Vinculin was used as loading control.

**C** Growth curves of p27KO H-Ras$^{V12}$ and p27KO H-Ras$^{V12}$ transformed fibroblasts, re-expressing hp27$^{WT}$, mp27$^{WT}$ or mp27$^{CK}$ proteins, as indicated. Data display the number of cells at 5 days from plating and represent the mean of three different experiments +/- SD.

**D** Soft agar assay of the indicated H-Ras$^{V12}$ transformed fibroblasts. Data represent the mean (+/-SD) of three different experiments performed in duplicate in which 8-10 fields/well were scored.

**E** Transwell migration assay of the indicated H-Ras$^{V12}$ transformed fibroblasts plated on transwell inserts coated with fibronectin and allowed to migrate up to 8 hours. Data represent the mean of two experiments performed in duplicate.

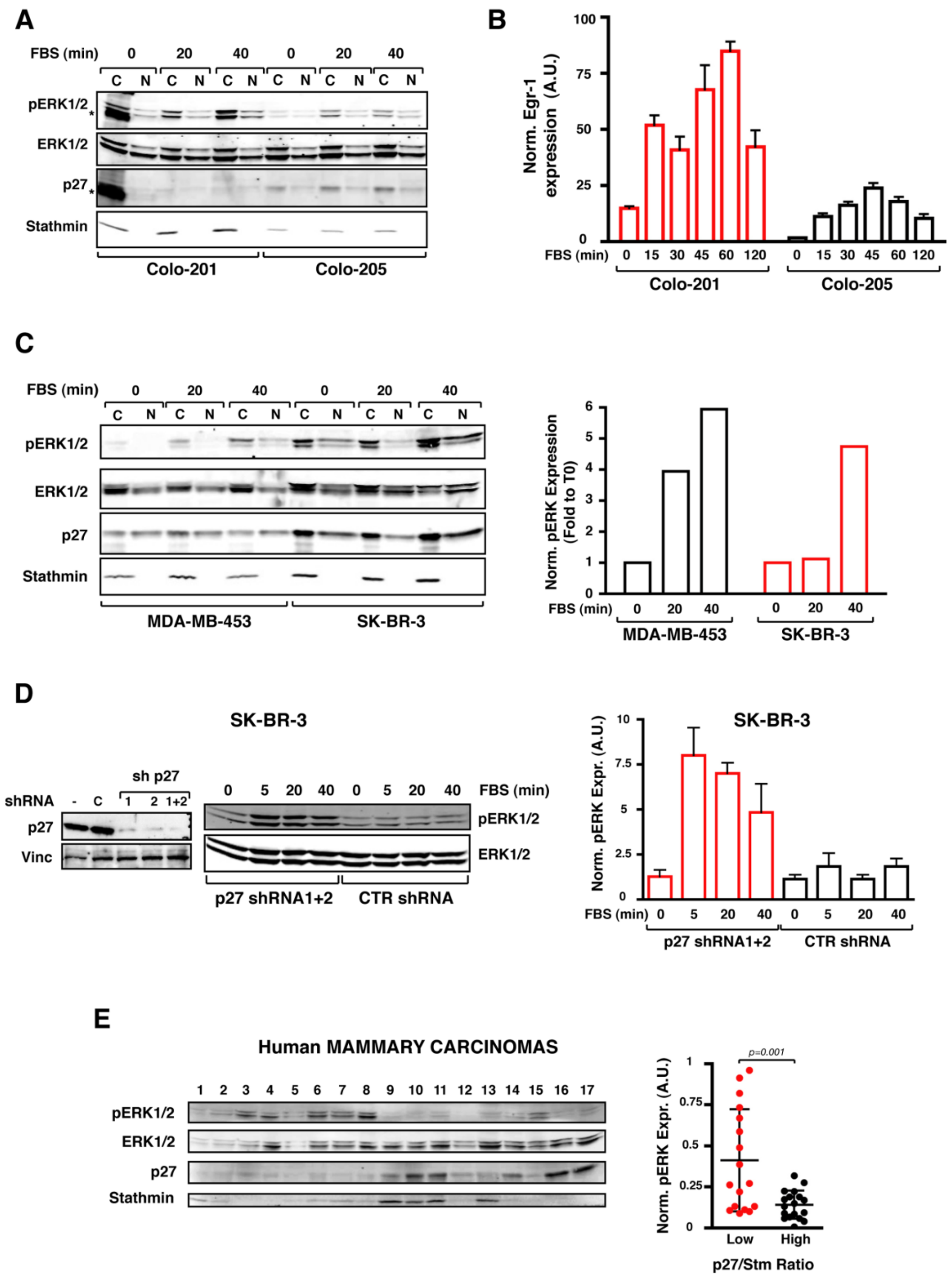


**Supplementary Figure S4.** *Low cytoplasmic p27/stathmin ratio is associated with hyper-activation of ERK1/2 pathway in human carcinomas and carcinoma-derived cell lines.*

**A** Western Blot analysis of ERK1/2 phosphorylation status, and p27 and stathmin expression levels in two human colon carcinoma cell lines (non B-Raf mutated), serum starved and then stimulated with complete medium (FBS) for the indicated

times. At each time point, cells were lysed to separate their nuclear (N) and cytoplasmic (C) fractions.

**B** qRT-PCR analysis of Egr-1 expression in Colo-201 and Colo-205 cells serum starved and then stimulated with complete medium (FBS) for the indicated times. Data were normalized using the expression of an housekeeping gene and expressed as fold increase over the level of time zero. A.U., arbitrary units. Error bars represent SEM

**C** Western Blot analysis of ERK1/2 phosphorylation status, and p27 and stathmin expression levels in two HER2 positive human breast carcinoma cell lines, serum starved and then stimulated with complete medium (FBS) for the indicated times. At each time point, cells were lysed to separate their nuclear (N) and cytoplasmic (C) fractions. In the right graph, the normalized ERK phosphorylation expressed as fold increase over the T0 (i.e. serum deprived cells) is reported.

**D** Western Blot analysis of ERK1/2 phosphorylation (right panel) in SK-BR-3 mammary carcinoma cell line, following silencing of p27 with specific shRNAs (left panel). Control shRNAs were used to exclude transduction-related non-specific effects. On the right, graph reports the quantification of ERK activation, normalized by total ERK expression in each corresponding lysate.

**E** Western Blot analysis of ERK1/2 phosphorylation, p27 and stathmin expression in the cytoplasmic fractions of 17 representative human mammary carcinoma samples (out of 37 analyzed). Vinculin was used as loading control. The right graph reports the quantification of the western blot analysis of ERK1/2 phosphorylation in cytoplasm of 37 human breast cancer specimens, as listed in Supplementary Table 1. Tumors were segregated in two groups, displaying either low or high p27/stathmin ratio, then a value of normalized phospho-ERK1/2 expression was assigned to each sample.

# Supplementary Materials and Methods

## *In vivo* experiments

Primary tumors were established by subcutaneous injection of $1x10^6$ H-Ras or K-Ras4B transformed fibroblasts (MEF-LTAg or 3T3) into female athymic nude mice (Harlan, 7-8 weeks of age). Only in the case of 129S2/Sv-derived H-Ras-transformed 3T3, $2x10^6$ cells were injected. Tumor growth has been monitored every other day and, 18-20 days (for the H-Ras transformed cells) or 26 days (for the K-Ras) after injection, animals have been sacrificed and tumors, blood and lungs collected for further analyses.

All animal experimentation was reviewed and approved by the CRO institutional Animal Care and Use Committee (OPBA), authorized by Italian Ministry of Health (authorization # 616/2015-PR) and strictly conducted according to the OPBA guidelines.

## Cell cultures

Primary wild type (WT), p27 knock-out (p27KO) and p27/stathmin double KO (DKO) mouse embryo fibroblasts (MEF) were prepared from embryos at day 13.5, according to standard procedures. The correct genotype of WT, p27KO and p27/stathmin DKO cells was determined by PCR, as described[6,7]. 3T3 fibroblasts were generated from primary MEFs, following the 3T3 immortalization protocol, as described[48]. 293T/17 (ATCC CRL-11268) and HEK 293 (ATCC CRL-1573) cells were used for the production of retroviral and adenoviral particles, respectively. 293T/17 were used also for the ubiquitination assay. 3T3 fibroblasts, MEF, 293 cells, MDA-MB-453 and SK-BR-3 human mammary adenocarcinoma cells and MES-SA, HS-913T, HT-1080 sarcoma cell lines, were all cultured in DMEM supplemented with 10% FBS (Sigma).

Colo-201 and Colo-205 human colorectal adenocarcinoma cells were cultured in RPMI-1640 supplemented with 10% FBS (Sigma).

**Generation of stable cell clones**

To generate stable cell clones of 3T3 fibrobasts, cells (passage ≤ 45) were transduced with H-Ras$^{V12}$ (gently provided by Dr. R. Baserga) and K-Ras4B$^{V12}$ (obtained from ADDGENE consortium, donated by Dr. T. Jacks) both cloned in pMSCV-hygro retroviral vector (Clontech).

To generate stable cell clones of MEFs, primary cultures of fibroblasts (at passage 3) were concomitantly transduced with SV40 Large TAg (gently provided by Dr. R. Maestro, as BamH1 insert, and then cloned in pMSCV-puro retroviral vector, Clontech) and pMSCV-Hygro-H-Ras$^{V12}$ or pMSCV-Hygro-K-Ras4B$^{V12}$.

Where indicated, human p27$^{WT}$ or p27$^{1\text{-}170}$ and mouse p27$^{WT}$ or p27$^{CK-}$ cDNAs have been retrovirally transduced in 3T3-p27KO-H-Ras$^{V12}$ clones. Mouse p27$^{WT}$ and p27$^{CK-}$ cDNAs were gently provided by Dr. Bruno Amati.

Clones and pools were selected in complete medium supplemented with 1.5 μg/ml Puromycin and/or 0.4 mg/ml Hygromycin. The stable expression of the different constructs was tested by Western Blot analysis of the target protein. Each experiment has been performed using at least two independent clones or pools.

**p27 silencing and transient overexpression.**

shRNAs have been delivered in SK-BR-3 mammary carcinoma cell line by lentiviral transduction, using MISSiON system (pLKO lentiviral vector, SIGMA). Briefly, 293FT cells were transfected with pLP1, pLP2, pLP/VSV-G (Invitrogen recombinant lentivirus producing system) plus pLKO-shRNA (sh1_TRCN0000039930, sh2_TRCN0000009856) by calcium phosphate protocol. After 48 and 72 hours,

conditioned medium containing lentiviral particles was harvested and used to transduce target cells.

For the transient overexpression of p27 in HT-1080, tetracycline-inducible recombinant adenoviruses were generated according to manufacturer's instructions (AdenoX Tet ON Expression System 2, Clontech) and used to transduce cells, as previously described[8]. Cells have been used 48-72 hours after transduction.

**Proliferation assays**

Proliferation assays include: growth curve experiments, using the Trypan Blue exclusion test and the MTS assay (Promega); cell cycle distribution using flow cytometry, BrdU incorporation assay and soft agar assays.

For growth curves, 0.5-1x$10^5$ cells/well (depending on the experiment) were seeded in 6-well plates in complete medium in triplicate. Fresh medium was added every other day. At the indicated times, cells were detached in trypsin-EDTA and counted by Trypan Blue exclusion test.

For MTS assay, 1,000 cells/well were seeded in 96-well plates in sextuplicate. Cell Titer 96-Aqueous Cell Proliferation Assay kit (Promega) was used for the detection of viable cells following the manufacturer's procedures.

Cell cycle distribution was analyzed by flow cytometry, under several culture conditions. Depending on the experiments, cells were analyzed in exponential growth, after serum deprivation (24 hours) and release in complete medium (10% FBS, different time points). Cells were collected and fixed in ice-cold 70% ethanol and stored at -20°C. Cells were then washed in PBS and resuspended in propidium iodide staining solution (50 µg/ml propidium iodide + 100 µg/ml RNase A, in PBS). Stained cells were subjected to flow cytometry analysis (FACS) with a FACScan or a FACSCalibur instrument (BD Biosciences). Distribution of cells in G1, S and G2/M

phases of the cell cycle was calculated using the WinMDI2.8 software.

To evaluate the anchorage-independent cell growth, H-Ras$^{V12}$ and K-Ras$^{V12}$ transformed cells ($5\times10^3$ or $12\times10^3$ depending on the experiments) were resuspended in 0.4% Low Melting Agarose (in DMEM-10% FBS) and quickly overlaid on a previously gelified 0.6% bottom agar (0.6% Low Melting Agarose in DMEM-10% FBS). The experiments were performed in six-well tissue culture plates, in triplicate. Fresh medium with 1.5 µg/ml Puromycin and/or 0.4 mg/ml Hygromycin was added to the wells every 3 days. Where indicated, FTI inhibitor was added in the top agar and in the medium at the concentration of 6 µM. On day 15, the number of colonies was counted in 10 random fields at 10X magnification.

**Motility assays**

For 3D-Matrigel™ evasion assay, cells ($7{,}5\text{x}10^5$/ml) were included in Matrigel™ (6 mg/ml, Becton Dickinson) drops and maintained 1 hour upside-down at 37°C. Then, complete medium was added and cell motility monitored by transmission microscopy, using a Nikon TS100/F microscope and images collected using a CCD camera (Leica). The evasion ability was estimated by measuring the distance covered by the cells from the drop edges, 5 days after inclusion. To perform this analysis, cells were stained with crystal violet and pictures were taken. After conversion from pixel to millimeters, the actual distance covered was calculated.

For transwell-based migration assay, bottom side of HTS Fluoroblok™ were coated with 20µg/ml fibronectin (Sigma) in carbonate buffer at 4°C overnight and then saturated with PBS 1% BSA for 2 hours at room temperature. Cells were labeled with DiI (Molecular Probes, Life Technologies) for 20 minutes at 37°C, then seeded on the Fluoroblok™ upper chamber and incubated at 37°C for the indicated times. Migration was monitored at different time-points by detection of fluorescence from the top (non-

migrated cells) and the bottom (migrated cells) sides of the membrane, using the computer-interfaced Infinite M1000 Pro (TECAN).

For the wound-healing assay, cells were grown to confluence in multiwell-plates and then scratched using a sterile tip. Pictures have been collected at different time points to evaluate the wound closure and the distance covered.

**Tissue samples**

A total of 17 sarcoma (leiomyosarcomas and fibromyosarcomas) and 37 breast tumor specimens were collected and diagnosed at Centro di Riferimento Oncologico (CRO) of Aviano (Italy), according to the World Health Organization (WHO) criteria. Sarcoma samples derived from primary (n=8) or metastatic samples (n=9) were and described in Baldassarre et al. Cancer Cell 2005. Breast cancer specimens derived from locally advanced primary tumors, as better specified in Supplementary Table 1. Proteins were extracted from frozen tissues, as described in Supplementary Materials.

**RNA extraction, RT-PCR from tissue samples and quantitative Real-Time PCR**

Isolation of RNA from cells, lung and circulating cells samples was performed using RNeasy-Mini Kit (QIAGEN), according to the manufacturer's instructions. Disruption of the tissue sample was achieved by grinding the frozen tissue thoroughly with lead blocks. The homogenization was performed passing the lysate at least 5 times through a blunt 23-gauge needle fitted to an RNase-free syringe. This approach was used to identify circulating tumor cells and/or metastatic cells. We want to highlight that although we extracted the RNAs from tissues and from circulating cells pellets and we have probably lost any circulating free nucleic acid in our preparations we cannot completely exclude that our approach could have also led to the amplification of some residual circulating RNAs.

RNA were then quantified and retro-transcribed with AMV Reverse transcriptase, according to manufacturer's instructions (Promega) and the obtained cDNAs were amplified with nested PCR, in order to evaluate the presence of ectopic cells in tissue samples.

The following primers were used:

pMSCVforward 5'-CCCTTGAACCTCCTCGTTCGACC-3',

pMSCVreverse 5'-GAGACGTGCTACTTCCATTTGTC-3',

H-RASforward 5'-GGATCCATGACGGAATATAAG-3'

H-RASreverse 5'-GAATTCTCAGGAGAGCACACA-3'

p27-170reverse 5'-GGATCCCTCGAGTGTTCTGTTGGCTCTTTT-3'.

Samples were normalized by amplification of 18S ribosomal subunit. PCR products were then resolved on 1% agarose gels stained with ethidium bromide.

For quantitative Real-time PCR, absolute expression of mouse or human c-Fos, Egr-1, Jun-B was evaluated by qRT-PCR using SYBR Green dye-containing reaction buffer (Power SYBR® Green PCR Master Mix 2X, Applied Biosystems).

The following primes (Sigma) were used:

mouse JUN-b FW: 5'-GCACTAAAATGGAACAGCCCTT-3'

mouse JUN-b RW: 5'-GGCTCGGTTTCAGGAGTTTG-3'

mouse EGR-1 FW: 5'-CCTTCCAGTGTCCAATCTGCA-3'

mouse EGR-1 RW: 5'-CTGGCAAACTTCCTCCCACA-3'

mouse c-FOS FW: 5'-TGGTGAAGACCGTGTCAGGA-3'

mouse c-FOS RW: 5'-GCAGCCATCTTATTCCGTTCC-3'

mouse GAPDH FW: 5'-TGAGGACCAGGTTGTCTCCT-3'

mouse GAPDH RW 5'-CCCTGTTGCTGTAGCCGTAT-3'

mouse 18s FW: 5'-GGACCAGAGGCAAAGCATTTGC-3'

mouse 18s RW: 5’-TCAATCTCGGGTGGCTGAACGC-3’

mouse PGK1 FW: 5’-TACCTGCTGGCTGGATGG-3’

mouse PGK1 RW: 5’-CACAGCCTCGGCATATTTCT-3’

human EGR-1 FW: 5’-TTTGCCAGGAGCGATGAAC-3’

human EGR-1 RW: 5’-CCGAAGAGGCCACAACACTT-3’

human SDHA FW: 5’-AGAAGCCCTTTGAGGAGCA-3’

human SDHA RW: 5’-CGATTACGGGTCTATATTCCAG-3’

human βACTIN FW: 5’-CCAACCGCGAGAAGATGA-3’

human βACTIN RW: 5’-CCAGAGGCGTACAGGGATAG-3’

human 18s FW: 5’-CTCAACACGGGAAACCTCAC-3’

human 18s RW: 5’-CGCTCCACCAACTAAGAACG-3’

Standard curves (10-fold dilution from $10^1$ to $10^{-4}$ attomoles) were prepared both for target genes and for housekeeping genes. The incorporation of the SYBR Green dye into the PCR products was monitored in real time using the Applied Biosystems ABI PRISM 7700 Sequence Detector, and the resulting threshold cycles (Ct) were computed. Ct values were converted into attomoles and the normalized target gene value was obtained by using at least two different housekeeping genes.

**Preparation of protein lysates, immunoprecipitation and immunoblotting**

Proteins were collected in indicated culture conditions, such as exponential growth, high confluence, starvation in serum free medium and after release in complete medium (10% FBS) or adhesion to Fibronectin (10 μg/ml, Sigma). Where indicated, cells have been also treated with the following compounds for the indicated time: FTI-276 (6 μM, Sigma), Y27632 (10 μM, Calbiochem), EGF (3 ng/ml, Invitrogen).. To extract total proteins from cells, at the indicated time points, cells were scraped on ice using cold NP40 lysis buffer (0.5% NP40; 50 mM HEPES pH 7; 250 mM NaCl; 5

mM EDTA; 0.5 mM EGTA, pH 8), supplemented with a protease inhibitor cocktail (Complete™, Roche) and 1 mM $Na_3VO_4$ (Sigma), 10 mM NaF (Sigma) and 1 mM DTT (Sigma). To extract total proteins from mouse organs or from tumor specimens, the same procedure was used, except that tissue disruption was first achieved by using the TissueLyser II (QIAGEN).

To perform the differential extraction of cytoplasmic and nuclear proteins, cells were resuspended in buffer A (10 mM HEPES pH 7.9, 0.1 mM EDTA pH 8, 0.1 mM EGTA pH 8, 10 mM KCl, 1 mM DTT), supplemented with the above listed inhibitors. Samples were incubated in ice for 15 minutes, then 0.5% NP40 was added and samples were centrifuged at max speed for 1 minute at 4°C. The supernatant, representing the cytoplasmic protein fraction, was collected. After 3 washes in 10% NP40, pellets were digested in buffer C (20 mM HEPES pH 7.9, 1 mM EDTA pH 8, 1 mM EGTA pH 8, 400 mM NaCl, 1 mM DTT plus all the inhibitors) and incubated in ice for 20 minutes. Samples were then centrifuged at max speed for 15 minutes at 4°C, to recover the supernatant, representing the nuclear protein fraction.

For immunoblot analysis, proteins were separated in 4-20% SDS-PAGE (Criterion Precast Gel, Biorad) and transferred to nitrocellulose membranes (GE Healthcare). Membranes were blocked with 5% dried milk in TBS-0.1% Tween20 or in Odyssey Blocking Buffer (Licor, Biosciences) and incubated at 4°C overnight with primary antibodies. Following 3x washes of 10 minutes in TBS-0.1% Tween20, membranes were incubated 1 hour at RT with horseradish peroxidase (HRP)-conjugated secondary antibodies (GE, Healthcare) for chemi-luminescent detection (ECL, GE Healthcare) or with IR-conjugated (Alexa Fluor680, Invitrogen and IRDye 800, Rockland) secondary antibodies for infrared detection (Odyssey Infrared Detection System, Licor). The Re-Blot Plus Strong Stripping Solution (Millipore) was used to

strip the membranes, when reblotting was needed.

Primary antibodies were purchased from *BD*: p27 (610242), stathmin/metablastin (611146); *Santa Cruz*: vinculin (sc-7649), cyclin B1 (sc-245), cyclin A (sc-751), ERK1 (sc-94), AKT (sc-1618), p27 (sc-527, sc-528), K-Ras (sc30), K-Ras 2B (sc521), H-Ras (sc-520), SV40 LTAg (sc147); *Cell Signaling*: pERK1/2 T202/204 (9101), pAKT S473 (9271), Myc-Tag (2276); *Sigma*: OP18/stathmin (O0138), Flag (A2220), HA (A2095); *Roche*: GFP (11814460001); *Calbiochem*: pan Ras (OP40).

**Ras Pull-Down**

3T3 fibroblasts transformed with H-Ras$^{V12}$ or K-Ras$^{V12}$,or HT1080 cells transduced with expressing Ad-green adenoviruses encoding or not (control) for p27WT or p27CK- proteins, were lysed as described above and 2 mg of protein were incubated with glutathione S-transferase (GST)-Raf bound to glutathione-Sepharose high performance (GE Healthcare) for 1 h at 4°C. After thorough washes, the samples were boiled for 10 minutes in Laemmli buffer to detach active GTP-bound Ras, loaded on 15% SDS-PAGE gels (Criterion; Bio-Rad) and then immunoblotted using anti-FLAG antibody.

**Immunofluorescence Analysis**

For immunofluorescence analyses tumor specimens tissues embedded in OCT were sectioned with a cryostat and fixed with 4% PFA for 10 minutes at RT. After washing in PBS, sections were immersed into 10 mM citrate buffer pH 6.0 and antigens retrieved by boiling in the microwave (550 W, 20 minutes). Samples were slowly cooled down to RT and specimens permeabilized with 0.4% Triton X-100 (Sigma-Aldrich) in PBS for 10 minutes. Samples were then blocked with 10% normal goat serum in PBS with 0.1% Triton X-100 for 20 minutes, incubated ON at RT with anti-Ki67 polyclonal antibody (Abcam, Cambridge, UK) followed by incubation with anti-

rabbit AlexaFluor® 488-conjugated (Invitrogen), and propidium iodide 3 µg/ml + RNase A 100 µg/ml for 1 hour. Images were acquired with a Leica TCS SP2 confocal system (Leica Microsystems Heidelberg, Mannheim, Germany), using the Leica Confocal Software (LCS).

For BrdU incorporation assay, cells were seeded on 12-well plates (BD, Falcon) containing coverslips (Menzel-Glaser, 12 mm) and were incubated in standard growth conditions for 1 hour with 10 µM BrdU (Roche). Then, cells were fixed in 4% PFA in PBS at room temperature (RT) and permeabilized in HCl 1,5 N for 30 minutes at 37°C. Coverslips were washed 2 times in Borate Buffer 0.1 M pH 8.5 and 2 times in PBS. Incubation with primary antibody anti-BrdU (Roche) was performed 1 hour at 37°C, then samples were washed in PBS and incubated with secondary antibody (anti-mouse Alexa Fluor488-conjugated, Invitrogen) for 1 hour at RT or 30 minutes at 37°C. Finally, nuclear staining with propidium iodide 3 µg/ml + RNase 100 µg/ml for 30 minutes at RT was performed and coverslips were mounted on glass slides with Mowiol 4-88 (Calbiochem).

**Statistical analysis**

In each experiment, sample sizes were chosen based on statistical power calculation. The computer software PRISM (version 4, GraphPad, Inc.) was used to make graphs and all statistical analyses. In all experiments, differences were considered significant when p was ≤0.05. Statistical analyses included paired and un-paired t-tests, Mann-Whitney un-paired t-test as appropriate and as described in each figure.

## Supplementary Table S1

| | TNM Classification | Hystopathologic Classification | Estrogen and Progesterone Status | Her2 Status |
|---|---|---|---|---|
| 1 | pT3N2 | IC | E+ Pg+ | Her2 2+ |
| 2 | pT2N0(sn) (3cm) | IDC | E+ Pg+ | Her2 - |
| 3 | pT3(m)N1a | ILC | E+ Pg+ | Her2 - |
| 4 | pT2N1a (4cm) | IDC | E+ Pg+ | Her2 1+ |
| 5 | pT2N1a (4cm) | IDC | E+ Pg+ | Her2 - |
| 6 | pT4bN0 | ILC | E+ Pg+ | Her2 - |
| 7 | pT3N1mi | IDC | E+ Pg+ | Her2 1+ |
| 8 | pT3N3a | ILC | E+ Pg- | Her2 - |
| 9 | pT2(m)N1a (3.5cm) | ILC | E+ Pg- | Her2 1+ |
| 10 | pT4bN2a | IDC | E+ Pg+ | Her2 1+ |
| 11 | pT2(m)N2a (4cm) | IDC | E+ Pg+ | Her2 1+ |
| 12 | pT2N1mi (3cm) | IDC | E+ Pg+ | Her2 1+ |
| 13 | pT2N0 (3cm) | MC | E+ Pg+ | Her2 1+ |
| 14 | pT2N2a | IDC | E+ Pg+ | Her2 3+ |
| 15 | pT2N1mi (3cm) | ILC | E+ Pg+ | Her2 - |
| 16 | pT2N0 (3cm) | IC | E- Pg+/- | Her2 1+ |
| 17 | pT2N1mi (3cm) | IDC | E- Pg- | Her2 3+ |
| 18 | pT3(m)N1a | IDC | E+ Pg+ | Her2 1+ |
| 19 | pT4bN3a | ILC | E+ Pg+ | Her2 1+ |
| 20 | pT3N2a | IDC | E- Pg- | Her2 1+ |
| 21 | pT2N0 (3cm) | IDC | E+ Pg- | Her2 1+ |
| 22 | pT3N1a | MC | E+ Pg+ | Her2 - |
| 23 | pT2N1a (dx) pT2N0 (sn) | IDC | E+ Pg- | Her2 3+ |
| 24 | pT2N3a (4.5cm) | IDC | E+ Pg- | Her2 2+ |
| 25 | pT2N1a (4.5cm) | ILC | E+ Pg+ | Her2 1+ |
| 26 | pT2(m)N2a | IDC | E+ Pg+ | Her2 - |
| 27 | pT2(m)N3a (3cm) | ILC | E+ Pg+ | Her2 1+ |
| 28 | pT4bNx (dx) pT2N3 (sn) | IDC | E+ Pg+ | Her2 2+ |
| 29 | pT4bN1a | IDC | E+ Pg+ | Her2 2+ |
| 30 | pT2N2 (4cm) | IDC | E- Pg- | Her2 1+ |
| 31 | pT4b(m)N3a | ILC | E+ Pg+ | Her2 1+ |
| 32 | pT3N3a | ILC | E+ Pg+ | Her2 3+ |
| 33 | pT4bN3a | IDC | E+ Pg- | Her2 - |
| 34 | pT2(m)N3a (3cm) | Multi LC | E+ Pg+ | Her2 - |
| 35 | pT2N1a (3.2cm) | IC | E+ Pg+ | Her2 - |
| 36 | pT2N1a (3cm) | IDC | E+ Pg+ | Her2 2+ |
| 37 | pT3N1a | IDC | E+ Pg+ | Her2 3+ |

IC, Invasive Carcinoma; IDC, Invasive Ductal Carcinoma; LC, Lobular Carcinoma; ILC, Invasive Lobular Carcinoma; MC, Mucinous Carcinoma; E, Estrogen Receptor; Pg, Progesterone Receptor;

**Breast Cancer Samples.** Specimens were diagnosed and collected at CRO of Aviano (Italy), according to the World Health Organization (WHO) criteria. TNM criteria have been used to classify the tumors.
